# Possible mitochondrial dysfunction in a patient with deafness, dystonia, and cerebral hypomyelination (DDCH) due to *BCAP31* Mutation

**DOI:** 10.1002/mgg3.1129

**Published:** 2020-01-17

**Authors:** Kenji Shimizu, Daiju Oba, Ryusuke Nambu, Manabu Tanaka, Eiji Oguma, Kei Murayama, Akira Ohtake, Koh‐ichiro Yoshiura, Hirofumi Ohashi

**Affiliations:** ^1^ Division of Medical Genetics Saitama Children’s Medical Center Saitama Japan; ^2^ Division of Gastroenterology and Hepatology Saitama Children’s Medical Center Saitama Japan; ^3^ Division of General Pediatrics Saitama Children’s Medical Center Saitama Japan; ^4^ Department of Radiology Saitama Children’s Medical Center Saitama Japan; ^5^ Department of Metabolism Chiba Children’s Hospital Chiba Japan; ^6^ Department of Pediatrics & Clinical Genomics Saitama Medical University Saitama Japan; ^7^ Department of Human Genetics Nagasaki University Graduate School of Biomedical Sciences Nagasaki Japan

**Keywords:** *BCAP31*, DDCH, mitochondrial dysfunction

## Abstract

**Background:**

Deafness, dystonia, and cerebral hypomyelination (DDCH) is an X‐linked disorder due to hemizygous mutations of *BCAP31*.

**Methods:**

We report an 8‐year‐old boy with DDCH who possibly accompanied mitochondrial dysfunction. Clinical evaluation, respiratory chain enzyme assay, and whole exome sequencing analysis were performed.

**Results:**

Mitochondrial dysfunction was suspected by respiratory chain enzyme assay on his cultured skin fibroblasts which showed significantly decreased complex I enzyme activity. Whole exome sequencing analysis revealed a recurrent *BCAP31* mutation (c.97C>T:p.Gln33*) which confirmed the diagnosis of DDCH for the patient.

**Conclusion:**

We speculate that mitochondrial dysfunction may be a feature in patients with DDCH.

## Graphical abstract

Deafness, dystonia, and cerebral hypomyelination (DDCH) is an X‐linked disorder due to hemizygous mutations of *BCAP31*. Mitochondrial dysfunction may be associated with DDCH.

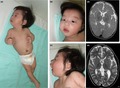

## 1 INTRODUCTION

Deafness, dystonia, and cerebral hypomyelination (DDCH; MIM#300475) is an X‐linked severe neurological disorder caused by hemizygous mutations of *BCAP31*. This condition was first defined in detail by Cacciagli et al.(2013) based on the observation of 11 affected male patients from unrelated three families through an attempt to identify mutations causing X‐linked intellectual disability by using next‐generation sequencing (Cacciagli et al., 2013).

Cardinal neurological features of these patients include severe motor and intellectual disabilities, sensorineural deafness, dystonia, and white matter hypomyelination and cerebral atrophy on brain MRI. Another unrelated patient with DDCH was reported who also showed features suggestive of mitochondrial encephalopathy such as bilateral increased signal intensity in globus pallidus on brain MRI (Albanyan, Al Teneiji, Monfared, & Mercimek‐Mahmutoglu, 2017). While the patient showed pleomorphic subsarcolemmal mitochondria collection on electron microscopy of muscle, respiratory chain enzyme activities of his muscle were normal.

All *BCAP31* mutations in four previously reported families were loss‐of‐function variants and inherited from carrier mothers. While all carrier females were intellectually normal, a mother was reported to have bilateral sensorineural hearing loss (Albanyan et al., 2017).

Here we report a patient with DDCH syndrome due to a recurrent, de novo, *BCAP31* mutation and with possible mitochondrial dysfunction as evidenced by decreased respiratory chain enzyme activity of complex I in the cultured skin fibroblast as well.

## 2 CLINICAL REPORT

The patient, an 8‐year‐old male, was born by cesarean section after 37‐week gestation to a 29‐year‐old primigravida mother and 30‐year‐old father, both Japanese, healthy and unrelated. His birth weight was 1,420 g (−3.6 *SD*), length 39.6 cm (−4.1 *SD*), and head circumference 28.5 cm (−2.5 *SD*). He was admitted to neonatal intensive care unit and given supplemental oxygen and phototherapy for jaundice for several days. A hearing test showed bilateral profound sensorineural hearing loss. Esotropia of the right eye was also noted.

**Figure 1:**
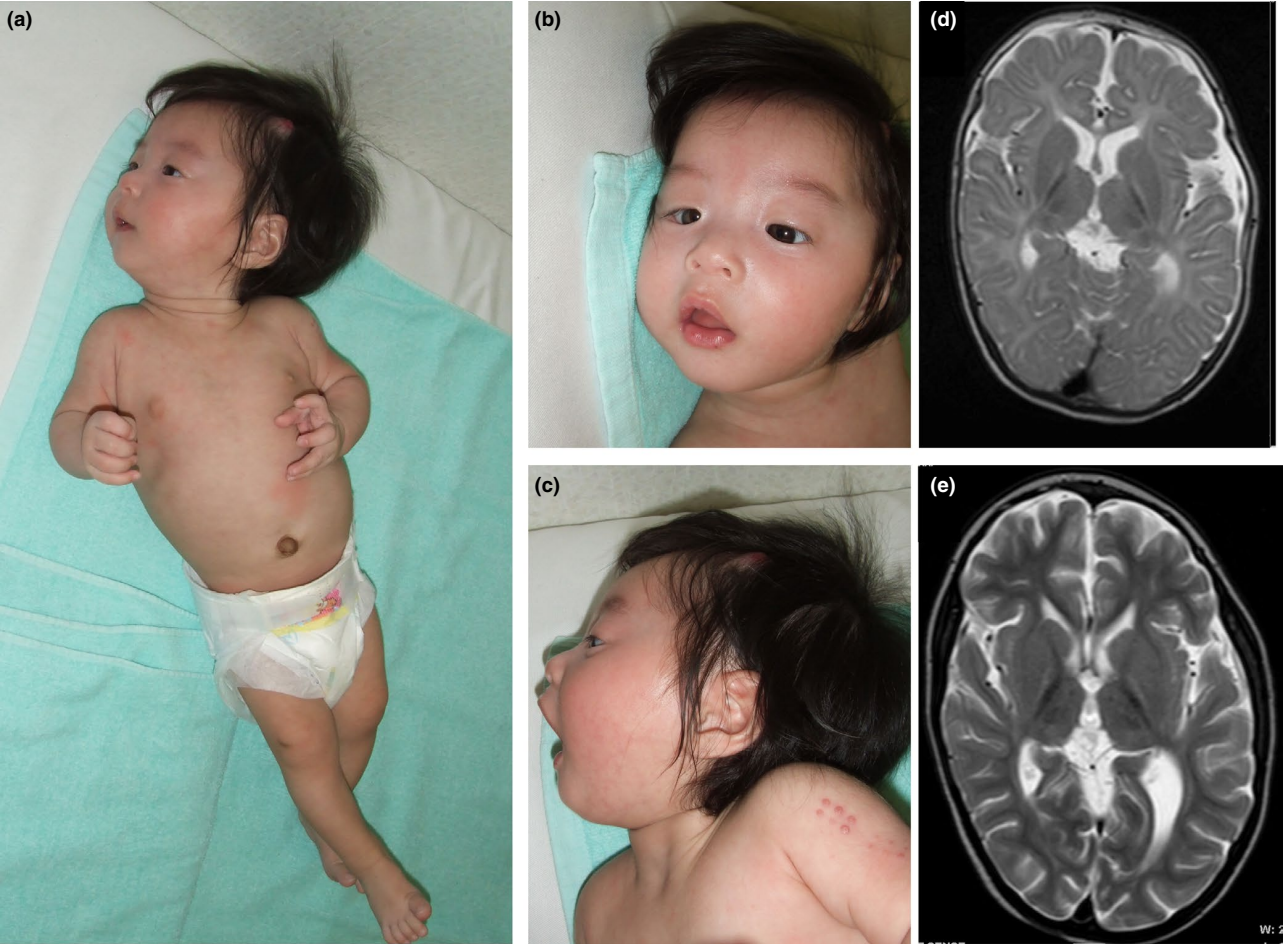
Phenotype of the patient at the age of 6 months (a‐c) and his MRI findings (d,e)

First seen by us at age 6 months, he showed dystonic movement characterized by sustained muscle contractions with lower limb twisting (Figure 1a). Other features noted were downslanted palpebral fissures, epicanthal folds, anteverted nares, a long and protruding philtrum, an open mouth with tented upper lip vermilion, and low‐set ears (Figure 1b,c). Strawberry hemangioma on the left parietal region, and cryptorchidism on the right side were also noted. He showed poor weight gain and marked developmental delay. He could control his head at age 2 years 6 months. He often showed elevated liver transaminase on febrile infections during early childhood.

At the age of 6 years, he developed high fever and involuntary movement associated with marked elevation of plasmic liver and biliary enzymes (AST 656 IU/L, ALT 148IU/L, and direct bilirubin up to 11.3mg/dl). On abdominal ultrasound, small intestinal necrosis associated with peritonitis with perforation was highly suspected, which was confirmed by an exploratory laparotomy, and then the necrotic region was surgically resected. At the age of 7 years and 9 months, he showed another episode of fever with elevated liver enzyme, which was found to be acute cholecystitis with gallbladder stones, and then he subsequently underwent cholecystectomy.

On brain MRIs at age of 1 year 8 months and 6 years 2 months, progressive brain atrophy especially in the white matter was apparent. Abnormalities also noted on both images were high intensities in bilateral thalamus and globus pallidus and in periventricular deep white matter on T2‐weighted images (Figure 1d,e).

Based on these MRI findings together with his clinical manifestations, we consider a possibility of his having mitochondrial dysfunction. Analyses by using his cultured skin fibroblasts revealed that activities of respiratory chain complexes I, II, III, and IV relative to citrate synthase were 19.8%, 32.7%, 115.4%, and 39.9%, respectively. The result showed possible mitochondrial dysfunction with reduced enzymatic activity of complex I.

Seen by us at 8 years 8 months, his height was 104 cm (−4.5 *SD*), body weight 14.77 kg (−2.3 *SD*), and head circumference 45.1 cm (−5.0 *SD*). He showed severe motor development delay and intellectual disability, and also showed orofacial and truncal hypotonia in contrast to spasticity of lower limbs with dystonic posture. His best developmental achievement was head control and rolling over to the right side.

## 3 GENETIC ANALYSIS

G‐banded chromosomal analysis revealed normal karyotype. No pathogenic copy number variants were identified by chromosomal microarray using Agilent Human Genome Microarray 244K (Agilent Technologies). Subsequently we performed trio‐based whole exome sequencing along the “Initiative on Rare and Undiagnosed Diseases in Pediatrics (IRUD‐P)” project of Japan, and identified a recurrent nonsense variant in *BCAP31*, c.97C>T:p.(Gln33*) (NM_001139441.1), in the patient. This variant was not detected in both of his parents. Mitochondrial DNA analysis by whole mitochondrion genome sequencing showed no abnormality associated with mitochondrial respiratory chain complex deficiencies.

Each genetic analysis was performed after obtaining written informed consent from the patient's parents, and the whole exome sequencing study through IRUD‐P project was approved by the institutional review board of our hospital.

## 4 DISCUSSION

We reported here a patient with DDCH due to *BCAP31* mutation associated with possible mitochondrial dysfunction. He had cardinal features of DDCH, including deafness, dystonia, severe developmental delay, and myelination abnormality, and in addition he showed brain MRI findings suggestive of mitochondrial encephalopathy such as cerebral atrophy and high signal intensity in thalamus and globus pallidus on T2‐weighed imaging, and then was diagnosed as having possible mitochondrial dysfunction with decreased mitochondrial respiratory chain enzymatic activity of complex I in the cultured skin fibroblast.

Prior to the identification of *BCAP31* pathogenic variants in patients with DDCH by Cacciagli et al.(2013), patients with *BCAP31* deficiency had been reported as Xq28 microdeletion involving *BCAP31* and its franking genes, including SLC6A8 (proximal to *BCAP31*) and *ABCD1* (distal to *BCAP31*) (Anselm et al., 2006; Calhoun & Raymond, 2014; Corzo et al., 2002; Iwasa et al., 2013; van de Kamp et al., 2015; Osaka et al., 2012). Features of DDCH such as deafness, dystonia, severe developmental delay, and myelination abnormality were almost consistently observed in these patients. Notably, codeletion of SLC6A8 with *BCAP31* might be at risk for developing mitochondrial dysfunction, and codeletion of *ABCD1* with *BCAP31* tends to be at risk for developing severe liver failure (Table 1).

**Table 1:** Clinical manifestations in patients with DDCH due to isolated *BCAP31* mutation and due to Xq28 microdeletion

Characteristics	Isolated *BCAP31* mutation					Xq28 microdeletion		
	Family 1 Cacciagli et al.	Family2 Cacciagli et al.	Family3 Cacciagli et al.	Albanyan et al.	Current Patient	Osaka et al. Anselm et al.	Corzo et al. Kamp et al. Iwasa et al.	Corzo et al. Kamp et al. Calhoun et al.
Genotype	c.194−2A > G	Exon 8 deletion	c.97C > T	c.533_536dup	c.97C > T	*BCAP31* and *SLC6A8* deletion	*BCAP31*and *ABCD1* deletion	*BCAP31, SLC6A8* and *ABCD1* deletion
Patient number	*n* = 2	*n* = 4	*n* = 1	*n* = 1	*n* = 1	*n* = 2	*n* = 4	*n* = 3
Age or Age at death	death at 13 and 24 years	death at 7 months−2 years except one living at 13 years	death at 3 years	living at 4.5 years	living at 8 years 8 months	one living at 9 years the other death at 8 years,	all death at 4–11 months	All death at 4–9 months
Developmental and cognitive status	no acquired motor milestones no cognitive sign	no acquired‐only head control except one sitting at 5 years	only head control no cognitive sign	no acquired motor milestones no cognitive sign	only head control and roll over, no speech	no acquired motor milestones	severe delay	no acquired motor milestones
Deafness	+	+ (*n* = 3)	**+**	**+**	**+**	**+**	+ (*n* = 3)	+ (*n* = 1)
Dystonia or Choreoathetosis	**+**	**+** (*n* = 3)	**+**	**+**	**+**	+	+	+
Brain MRI	hypomyelination cerebral and cerebellar atrophy	hypomyelination	hypomyelination cerebral atrophy	high SI in globus pallidus	high SI in thalamus and globus pallidus cerebral atrophy	hypomyelination, high SI in basal ganglia or globus pallidus, cerebral and cerebellar atrophy	hypomyelination, white matter abnormalities	hypomyelination
Hepatobiliary abnormality	NM^a^	NM^a^	NM^a^	elevated transaminase	cholecystitis with gallbladder stones	Elevated transaminase	cholestasis fibrosis liver failure	cholestasis liver failure
Mitochondrial respiratory chain activity	NM	NM	normal	normal	significant decrease in complex I	significant decrease in complex I (*n* = 1)	NM	NM
Other mitochondrial abnormality	−	−	−	pleomorphic subsarcolemmal mitochondria	NE	NM	NM	NM

Note

+, the feature present; −, the feature absent; SI, signal intensities; NM, the feature not mentioned; NE, the feature not evaluated.

a The feature was not mentioned according to each family, but four patients among all the families reported by Cacciagli displayed mild elevated transaminase.

In terms of mitochondrial dysfunction, Cacciagli et al. (2013) showed altered ER morphology and disorganization of the Golgi apparatus in fibroblasts of patients with DDCH, while the mitochondrial respiratory chain activities in the muscle of a patient in the family were normal and the mitochondrial network displayed a normal morphology in all affected fibroblasts. Another patient with DDCH reported by Albanyan et al. (2017) showed manifestations suggestive for mitochondrial dysfunction such as  choreoathetosis, increased signal intensity in the globus pallidus on MRI images, and pleomorphic subsarcolemmal mitochondria collection on electron microscopy in his biopsied muscle, while mitochondrial complex chain activities were not decreased as was the patient reported by Cacciagli et al. (2013). (Table 1).

Although mitochondrial dysfunction is etiologically extremely heterogeneous (Kohda et al., 2016; Ohtake et al., 2014), it could be classified into primary mitochondrial disease and secondary mitochondrial dysfunction: the former due to germline mutations in mitochondrial or nuclear DNA genes encoding electron transport chain proteins in oxidative phosphorylation (oxphos), and the latter due to variable pathologic processes not involving oxphos genes (Niyazov, Kahler, & Frye, 2016). It is noteworthy that secondary mitochondrial dysfunction has recently been increasingly recognized to co‐occur in association with various disorders including neuromuscular (such as spinal muscular atrophy) and neurodegenerative (such as Alzheimer disease) disorders (Niyazov et al., 2016). Although the pathophysiology underlying the mitochondrial dysfunction seen in such disorders are not fully understood, of note is a suggestion that dysfunction of mitochondrial associated membranes (MAMs) has been involved in these disorders. MAMs are implicated in functions of ER‐mitochondrial crosstalk including intracellular calcium homeostasis, lipid metabolism, mitochondrial fission, autophagosome formation, and apoptosis progression through proper mitochondria‐ER tethering (Liu & Zhu, 2017). Since BAP31 is involved in this ER‐mitochondrial tethering/crosstalk by interaction with Fission 1 homolog, it might be reasonable to consider that BAP31 deficiency possibly results in secondary mitochondrial dysfunction (Iwasawa, Mahul‐Mellier, Datler, Pazarentzos, & Grimm, 2011).

In conclusion, mitochondrial dysfunction may be a feature with DDCH due to *BCAP31* mutation, and patients with DDCH should be carefully evaluated for mitochondrial dysfunction.

## CONFLICT OF INTEREST

The authors have no conflict of interest to declare.

## References

[mgg31129-bib-0001] Albanyan, S. , Al Teneiji, A. , Monfared, N. , & Mercimek‐Mahmutoglu, S. (2017). BCAP31‐associated encephalopathy and complex movement disorder mimicking mitochondrial encephalopathy. American Journal of Medical Genetics. Part A, 173(6), 1640–1643. 10.1002/ajmg.a.38127 28332767

[mgg31129-bib-0002] Anselm, I. M. , Alkuraya, F. S. , Salomons, G. S. , Jakobs, C. , Fulton, A. B. , Mazumdar, M. , … Marsden, D. (2006). X‐linked creatine transporter defect: A report on two unrelated boys with a severe clinical phenotype. Journal of Inherited Metabolic Disease, 29(1), 214–219. 10.1007/s10545-006-0123-4 16601897 PMC2393549

[mgg31129-bib-0003] Cacciagli, P. , Sutera‐Sardo, J. , Borges‐Correia, A. , Roux, J.‐C. , Dorboz, I. , Desvignes, J.‐P. , … Villard, L. (2013). Mutations in BCAP31 cause a severe X‐Linked phenotype with deafness, dystonia, and central hypomyelination and disorganize the Golgi apparatus. American Journal of Human Genetics, 93(3), 579–586. 10.1016/j.ajhg.2013.07.023 24011989 PMC3769969

[mgg31129-bib-0004] Calhoun, A. R. U. L. , & Raymond, G. V. (2014). Distal Xq28 microdeletions: Clarification of the spectrum of contiguous gene deletions involving ABCD1, BCAP31, and SLC6A8 with a new case and review of the literature. American Journal of Medical Genetics, Part A, 164(10), 2613–2617. 10.1002/ajmg.a.36661 25044748

[mgg31129-bib-0005] Corzo, D. , Gibson, W. , Johnson, K. , Mitchell, G. , LePage, G. , Cox, G. F. , … Steinberg, S. J. (2002). Contiguous deletion of the X‐linked adrenoleukodystrophy gene (ABCD1) and DXS1357E: A novel neonatal phenotype similar to peroxisomal biogenesis disorders. American Journal of Human Genetics, 70(6), 1520–1531. 10.1086/340849 11992258 PMC419992

[mgg31129-bib-0006] Iwasa, M. , Yamagata, T. , Mizuguchi, M. , Itoh, M. , Matsumoto, A. , Hironaka, M. , … Shimozawa, N. (2013). Contiguous ABCD1 DXS1357E deletion syndrome: Report of an autopsy case. Neuropathology, 33(3), 292–298. 10.1111/j.1440-1789.2012.01348.x 22994209

[mgg31129-bib-0007] Iwasawa, R. , Mahul‐Mellier, A. L. , Datler, C. , Pazarentzos, E. , & Grimm, S. (2011). Fis1 and Bap31 bridge the mitochondria‐ER interface to establish a platform for apoptosis induction. EMBO Journal, 30(3), 556–568. 10.1038/emboj.2010.346 21183955 PMC3034017

[mgg31129-bib-0008] Kohda, M. , Tokuzawa, Y. , Kishita, Y. , Nyuzuki, H. , Moriyama, Y. , Mizuno, Y. , … Okazaki, Y. (2016). A comprehensive genomic analysis reveals the genetic landscape of mitochondrial respiratory chain complex deficiencies. PLoS Genetics, 12(1), 1–31. 10.1371/journal.pgen.1005679 PMC4704781 26741492

[mgg31129-bib-0009] Liu, Y. , & Zhu, X. (2017). Endoplasmic reticulum‐mitochondria tethering in neurodegenerative diseases. Translational Neurodegeneration, 6(1), 1–8. 10.1186/s40035-017-0092-6 28852477 PMC5567882

[mgg31129-bib-0010] Niyazov, D. M. , Kahler, S. G. , & Frye, R. E. (2016). Primary mitochondrial disease and secondary mitochondrial dysfunction: importance of distinction for diagnosis and treatment. Molecular Syndromology, 7(3), 122–137. 10.1159/000446586 27587988 PMC4988248

[mgg31129-bib-0011] Ohtake, A. , Murayama, K. , Mori, M. , Harashima, H. , Yamazaki, T. , Tamaru, S. , … Okazaki, Y. (2014). Diagnosis and molecular basis of mitochondrial respiratory chain disorders: Exome sequencing for disease gene identification. Biochimica Et Biophysica Acta (BBA) ‐ General Subjects, 1840(4), 1355–1359. 10.1016/j.bbagen.2014.01.025 24462578

[mgg31129-bib-0012] Osaka, H. , Takagi, A. , Tsuyusaki, Y. U. , Wada, T. , Iai, M. , Yamashita, S. , … Matsumoto, N. (2012). Contiguous deletion of SLC6A8 and BAP31 in a patient with severe dystonia and sensorineural deafness. Molecular Genetics and Metabolism, 106(1), 43–47. 10.1016/j.ymgme.2012.02.018 22472424

[mgg31129-bib-0013] van de Kamp, J. M. , Errami, A. , Howidi, M. , Anselm, I. , Winter, S. , Phalin‐Roque, J. , … Salomons, G. S. (2015). Genotype‐phenotype correlation of contiguous gene deletions of SLC6A8, BCAP31 and ABCD1. Clinical Genetics, 87(2), 141–147. 10.1111/cge.12355 24597975

